# Multipotent Mesenchymal Stromal Cells in Rheumatoid Arthritis and Systemic Lupus Erythematosus; From a Leading Role in Pathogenesis to Potential Therapeutic Saviors?

**DOI:** 10.3389/fimmu.2021.643170

**Published:** 2021-02-24

**Authors:** Jehan J. El-Jawhari, Yasser El-Sherbiny, Dennis McGonagle, Elena Jones

**Affiliations:** ^1^Department of Biosciences, School of Science and Technology, Nottingham Trent University, Nottingham, United Kingdom; ^2^Department of Clinical Pathology, Faculty of Medicine, Mansoura University, Mansoura, Egypt; ^3^Faculty of Medicine and Health, Leeds Institute of Rheumatic and Musculoskeletal Medicine, University of Leeds, Leeds, United Kingdom; ^4^The National Institute for Health Research Leeds Biomedical Research Centre, Chapel Allerton Hospital, Leeds, United Kingdom

**Keywords:** Multipotent Mesenchymal Stromal Cells, rheumatoid arthritis, systemic lupus erythematosus, autoimmunity, immune therapy, immunomodulaion

## Abstract

The pathogenesis of the autoimmune rheumatological diseases including rheumatoid arthritis (RA) and systemic lupus erythematosus (SLE) is complex with the involvement of several immune cell populations spanning both innate and adaptive immunity including different T-lymphocyte subsets and monocyte/macrophage lineage cells. Despite therapeutic advances in RA and SLE, some patients have persistent and stubbornly refractory disease. Herein, we discuss stromal cells' dual role, including multipotent mesenchymal stromal cells (MSCs) also used to be known as mesenchymal stem cells as potential protagonists in RA and SLE pathology and as potential therapeutic vehicles. Joint MSCs from different niches may exhibit prominent pro-inflammatory effects in experimental RA models directly contributing to cartilage damage. These stromal cells may also be key regulators of the immune system in SLE. Despite these pro-inflammatory roles, MSCs may be immunomodulatory and have potential therapeutic value to modulate immune responses favorably in these autoimmune conditions. In this review, the complex role and interactions between MSCs and the haematopoietically derived immune cells in RA and SLE are discussed. The harnessing of MSC immunomodulatory effects by contact-dependent and independent mechanisms, including MSC secretome and extracellular vesicles, is discussed in relation to RA and SLE considering the stromal immune microenvironment in the diseased joints. Data from translational studies employing MSC infusion therapy against inflammation in other settings are contextualized relative to the rheumatological setting. Although safety and proof of concept studies exist in RA and SLE supporting experimental and laboratory data, robust phase 3 clinical trial data in therapy-resistant RA and SLE is still lacking.

## Introduction

The original description of multipotent mesenchymal stromal cells, previously known as mesenchymal stem cells (MSCs) that represented tissue-resident clonogenic stromal cells with multilineage osteogenic, chondrogenic, adipogenic and potentially other stromal lineage differentiation capacities emphasized their role in skeletal structure and integrity (1, 2). However, MSCs were later found to be capable of crosstalk with the immune system and have multifaceted additional immunomodulatory functions (3). Strong evidence of research over several years have shown that there are two-way interactions between MSCs and immune cells/factors (4, 5). The best characterized and popular MSCs for immunomodulatory therapies include bone marrow (BM)-MSCs, umbilical cord (UC)-MSCs and adipose-derived (AD)-MSCs. Whilst there is great interest in the use of MSCs for tissue regeneration and repair, the focus herein is in their less well-understood role in tissue homeostasis and immune regulation and on the attempts to translate this into novel immunomodulatory therapy strategies.

Major two-way communication exists between MSCs and immune cells. Cytokines exclusively derived from immune cells such as IL-22 can affect the survival, proliferation, and differentiation function of MSCs (6). Conversely, MSCs can initiate powerful contact-dependent and independent anti-inflammatory cascades that terminate immune responses in healthy condition (5). In contrast to this, MSCs can produce pro-inflammatory effects in pathological tissue environments and drive pathology (4, 7). In the context of RA, the cross-talk between MSCs and immune cells resident in the synovium presents a particular interest.

While MSCs were defined in 2006 as tripotential, plastic adherent cells with a characteristic surface phenotype according to International Society for Cell & Gene Therapy (ISCT) position statement (1), synovium-derived MSCs were first described in 2001 as clonogenic cells with the same ability to proliferate in culture and to have trilineage differentiation potential at the single-cell level (8). Subsequently, the typical surface phenotype of synovium-derived MSCs has been confirmed by flow cytometry (9). In contrast to synovium-derived MSCs, fibroblast-like synoviocytes terminology refers to a culture-expanded fibroblast population from synovium that has not been confirmed to have all MSC characteristics according to the strict ISCT criteria (9). A recent analysis of the literature revealed that both synovium-derived MSCs and fibroblast-like synoviocytes have similar fibroblastic morphology and no distinctive features clearly discriminating between both populations in terms of cell surface markers, differentiation potential or immunomodulatory functions (10, 11). Using the terminology of synovium-derived MSCs and fibroblast-like synoviocytes is likely to refer to the same cell population however, there is no complete agreement (12, 13) and further investigation is needed to clarify the relationship between these cells. Here, we refer to synovial multipotent mesenchymal stromal cells as “MSCs,” but we use “fibroblast-like synoviocytes” for describing specific studies that used this terminology.

This review will focus on the interesting crosstalk between MSCs and immune cells and how this crosstalk impacts inflammatory mediators in two autoimmune/inflammatory joint diseases that both exhibit joint predilections; rheumatoid arthritis (RA) and systemic lupus erythematosus (SLE). We focus on these diseases in the experimental model settings and on preliminary clinical trials data emerging in humans where proof of concept preclinical studies showing systemic MSC infusion may be associated with beneficial impacts. Consolidating this knowledge is essential to understand further the emerging field of MSC use for immune homeostasis restoration and to facilitate the development and optimization of therapeutic methods, particularly for resistant rheumatologic autoimmune disease that is refractory to all existing therapies.

## Pathogenesis of RA and SLE

The autoimmune diseases are self-directed inflammatory disorders characterized by progressive tissue destruction and a loss of tissue function if not adequately treated (14). Both RA and SLE are more common in females than males, but the ratio is exaggerated in SLE, where it is up to 10-fold more common in females (15). Autoantibody positive RA is typically progressive whereas SLE course is usually characterized by remission and relapse phases (16). SLE and RA have several shared genetic associations such as MHC-Class II genes and numerous genetic polymorphisms in genes linked to innate and adaptive immune cell function (17, 18).

Both RA and SLE are characterized by the presence of autoantibodies that predate clinical disease. The pathogenesis of SLE is particularly highlighted by over-activation of B-lymphocytes with excess production of autoantibodies and typically leads to multi-organ inflammation including skin, joint causing lupus arthritis (LA) and internal organs including the kidneys (18). In RA, the production of autoantibodies against citrullinated proteins in the joints and elsewhere is associated with the immune complex formation and pro-inflammatory cytokine production including TNF-α and IL-6 and this orchestrates extensive tissue damage. Although RA is much more joint centric than SLE, it is also viewed as a systemic disease and heterogeneous innate and adaptive mechanism may contribute to resistant disease where therapy remains suboptimal (19).

Despite differences in some molecular mechanisms, both RA and SLE are commonly characterized by the loss of peripheral immune tolerance that encompasses reduced T-reg cells in both settings (20, 21). Additionally, increased immune cell numbers and activation, especially in target end organs and excess production of cytokines and other immune mediators that culminates in chronic inflammation are common for both diseases (18). Given that a subgroup of RA and SLE cases are both refractory to all available conventional therapies, a need for experimental new therapy exists. Application of MSCs is one putative cell therapy strategy and has clinical data in the public domain for both the therapy of resistant RA and resistant SLE.

## MSC Function in Healthy Joints

When initially studied in the laboratory, MSCs were described as colony-forming fibroblasts with the capability to undergo massive clonal expansion (22). As mentioned, *in vitro* expanded MSCs have been characterized according to the ISCT criteria. These criteria include plastic adherence, lack of expression of hematopoietic lineage markers CD45, CD34, CD14 or CD11b, CD79 alpha, CD19, and HLA-DR, but the expression of surface molecules CD90, CD73, CD105 (1). The surface molecule CD271 is also considered as a distinctive marker for uncultured bone marrow (BM) and bone MSCs (23), which are most common and best-characterized MSCs within musculoskeletal tissues (24). Although not the focus of this article, MSCs are a reservoir of cells for the maintenance of subchondral bone, adipose tissue, ligaments, menisci, the synovial membrane and the synovial fluid homeostasis (4, 25). Interestingly, these MSCs are capable of migration toward the damaged areas e.g., MSCs can migrate from subchondral BM into the joint cavity as detected in underlining layers of proliferating synovial tissue (26), also MSCs were shown to be involved in the synovial hyperplasia in a mouse model of joint surface injury (27). Migratory chondroprogenitors have been found in damaged osteoarthritic cartilage (28), which could, in principle, originate from MSCs migrating upwards from the subchondral bone (29). At the synovio-entheseal complexes, populations of BM cells may theoretically have access to joint cavity via channels wide enough to facilitate MSCs passage from the subchondral bone plate (30). So, under normal condition, there may be potential for loco-regional migration of MSCs from different joint and bone MSC niches toward repair responses and possibly for immunomodulatory responses, but the latter is not well-studied.

A lesser appreciated role for MSCs comes from their immunomodulatory capabilities which have been well-reviewed elsewhere (5, 31, 32). Of note, most of the work pertaining to MSC immunomodulation in RA and SLE was conducted using culture expanded MSCs, but we have recently shown a potential immunosuppression capacity of uncultured MSCs in cancellous bones (33). Pro-inflammatory cytokines, IFN-γ TNF-α and IL-1 can induce or enhance the immunosuppressive capabilities by MSCs, a process that is termed “licensing” whereby pro-inflammatory cytokine treatment of MSCs increases their immunomodulatory capacity (4). Licensed MSCs can inhibit immune cells via soluble mediators; TGF-β, indoleamine 2,3-dioxygenase (IDO), inducible Nitric oxide synthases (iNOS), prostaglandin E2 (PGE2), IL-1 receptor antagonist (IL-1Ra) and tumor necrosis factor-inducible gene 6 (TSG6) (34–36). It must be pointed out that although the immunomodulatory function of MSCs was first reported in relationship to bone marrow MSCs (37), this property likely extends to other fibroblastic cells including synovial fibroblasts (38). It has also been reported that both synovium-derived MSCs and fibroblast-like synoviocytes possess similar immunomodulatory capacity by suppressing T cell proliferation via IDO and TGF-β -dependent mechanisms (39). Therefore, considering the immunomodulatory function of fibroblast-like synoviocytes alongside that of synovium-derived MSCs appears relevant, particularly as more data have emerged in this field recently (40).

Cell to cell contact is another important mechanism of MSC-mediated immunosuppression. Co-culture experiments between MSCs and NK cell lines have shown a change in the NK granule polarization (41). Additionally, MSCs can support neutrophil survival via a surface intercellular adhesion molecule 1 (ICAM-1)-dependent mechanism (42). Cellular interactions between MSCs and adaptive immune cells have also been reported. MSCs can suppress naïve T cell and memory T cell via ICAM-1 and vascular cell adhesion molecule 1 (VCAM-1) interactions (43). Similarly, MSCs can induce the Notch1/Forkhead box P3 (FOXP3) pathway in CD4+T cells and increase the percentage of CD4+CD25 FOXP3+cells (44). One of the surface proteins expressed on MSCs galectin-1 was linked to MSC-mediated immunosuppression of T lymphocytes (45). In addition to T cells, MSCs have been shown to increase the survival of quiescent B cells in cell-cell contact context (46) by activating p38 mitogen-activated protein kinase (MAPK) pathways (47). Therefore, it seems likely that the synovial MSCs not only contribute to normal joint tissue integrity and structure, but also have immunomodulatory characteristics qualifying their potential role in healthy joint homeostasis as well as RA or LA pathogenesis.

Outside the joint, MSCs and non-MSC stromal cells also exhibit similar immunomodulatory capabilities. For example, skin fibroblasts exert virtually the same immunomodulatory effects as BM-MSCs (48). An interesting aspect of the use of culture-expanded MSCs as effector cells for immunomodulation in RA is that these cells are given intravenously. However, there is little evidence for the physiological circulation of MSCs in humans, including patients with RA (49), which will be discussed further in this review.

## Altered MSC Functions in RA and SLE Joints

The intrinsic immunosuppressive role of MSCs within the joint may contribute to maintaining immune homeostasis within a healthy joint, while the opposite scenario could happen in the pathological microenvironment. As mentioned above, various stromal cell populations derived from synovial tissues have been assigned different terminologies. Fibroblast-like synoviocytes and Type B synoviocytes represent essentially the same cell type with the ability to produce proteoglycans, cytokines, arachidonic acid metabolites, and metalloproteinases (10). However, the term “fibroblast-like synoviocytes” is more commonly used to describe the pathogenic role of RA intimal fibroblasts in destructive joint inflammation unlike “synovial fibroblasts” that broadly defines all synovial-derived cells (50). All synovial fibroblasts and synovium-derived MSCs have similar morphology of spindle-shaped cells (51), but it has been proposed that these fibroblasts can be the aged form of MSCs probably with altered immune and repair related functions (12). Another suggestion was that MSCs and synovial fibroblasts could represent dissimilar functional stages of the same stromal cell lineage (13). Importantly, a recent study has identified and described two anatomically different subsets of fibroblasts accountable for mediating either inflammation or tissue damage in arthritis murine models (40). The synovial sub-lining cells, FAPα^+^ THY1^+^ fibroblasts were found to mediate inflammation in murine arthritis. In contrast, FAPα^+^ THY1^−^ located in synovial lining layer were linked to destructive bone changes and cartilage damage (40).

Although immune cells are key orchestrators of RA and SLE, there is evidence that fibroblast-like synoviocytes are also contributing to joint disease pathogenesis (50). When MSCs were harvested from RA patients, *in vitro* immunosuppression was evident, however, it seems that the microenvironment of RA joint reduced efficacy of MSCs to control the exaggerated immune response in this disease (4, 52). Citrullinated fibrinogen (a prominent auto-antigen in RA) can affect BM-MSC function by promoting IL-6, IL-8, CCL2 expression and reducing IDO via the involvement of toll-like receptor 4 (TLR-4) and NFκB pathway (53). Interestingly, while stimulation of TLR4 expressed on MSCs causes the production of pro-inflammatory cytokines IL-6 and IL-8, TLR3 engagement induced immunosuppressive MSCs to produce IDO (54, 55). Another defect reported in RA BM-MSCs is A20 downregulation leading to higher IL-6 expression levels, further contributing to RA progression (56). A20 is an anti-inflammatory protein that prevents NF-kB hyperactivation following excess exposure of cells to TNF-α (57). In summary, variable mechanisms could induce pro-inflammatory function of MSCs contributing to RA pathogenesis.

There are reports of MSC immunosuppressive functions alterations in SLE. It has been reported that BM-MSCs from SLE patients had less IDO production than healthy MSCs when both MSCs were licensed by IFN-γ (58). Thus, the modulation of IDO activity could be potentially useful to reinstate the functions of SLE BM-MSCs. Other studies showed lower expression of immunosuppressive cytokines such as TGF-β and IL-10 by SLE BM-MSCs because of abnormal activation of several JAK-STAT, p53/p21, PTEN/Akt, PI3K/Akt, and Wnt/beta-catenin signaling pathways (59–61). Despite evidence of less immunosuppression, the pro-inflammatory effect of SLE MSCs and mechanisms causing these changes are not fully clear.

Besides immunomodulatory alterations, several *in vitro* studies showed that SLE MSCs have a tendency toward senescence. Increased ROS indicating endoplasmic reticulum stress was linked to the senescence of SLE MSCs as detected by electron microscopy (62). Also, induced levels of senescence-related genes that block the cell cycle in MSCs from SLE patients were associated with limited proliferation and cell morphology showing deeply stained nucleus, and disordered cytoskeletal organization (63). These biological characters seemed to be linked to mitochondrial antiviral signaling protein (MAVS) that induces an IFN-β and ROS expression in SLE MSCs (64, 65). IFN-β is involved in cell senescence via p53 transcriptional and functional alterations of P53 (66). Senescence-associated cytokines, including IL-6, IL-8, and granulocyte–macrophage colony-stimulating factor (GM-CSF), significantly increased at the gene expression levels in SLE MSCs (64). Furthermore, increased susceptibility to death was suggested for SLE MSCs due to significantly low levels of an anti-apoptotic marker, Bcl-2 (67) and where SLE BM-MSCs were reported to have a normal karyotype (68).

In summary, the functions and proliferative capacity of MSCs are changed in RA and SLE either due to intrinsic or extrinsic reasons but all associated with chronic inflammation. Multiple subsets of immune cells are present in the autoimmune arthritis milieu, and their interactions with MSCs are discussed below.

## MSCs Interaction with Key Immune Cell Players in RA and SLE

### DCs and MSCs

In normal condition, MSCs have inhibitory effects on dendritic cells (DCs). MSCs can suppress the monocyte differentiation into DCs or switch monocyte differentiation into macrophages rather than antigen-presenting DCs via several mechanisms including IL-6, TSG-6, and COX-2/PGE2 (69–71). Furthermore, MSCs can inhibit the maturation, migration and antigen presentation of DCs as murine DCs co-cultured with murine MSCs also found to induce more T-reg cells (72). Additionally, the production of IL-1Ra via MSCs can inhibit the production of IL-1β, IL-6, and IL-23 by DCs in co-cultures (73).

Monocyte-derived DCs can acquire the phenotype of myeloid-derived suppressor cells (MDSC) via the growth-regulated oncogene (GRO) chemokines produced by human MSCs. These DCs were found to have high secretion of IL-10 and IL-4 but low expression of IL-12 and IFN-γ (74). Additionally, human MSCs were reported to promote the expansion of MDSCs expressing arginase-1 (ARG-1) and iNOS through hepatocyte growth factor bractice HGF-related mechanism (75). Another mechanism by which MSCs can support the survival of MDSCs is exosomes produced by MSCs that induce the anti-apoptotic proteins Bcl-xL and Mcl-1 as seen in tumor cells (76). The variety of mechanisms by which MSCs inhibit DC function indicate that MSCs might have an underestimated role of keeping immune hemostasis (mainly suppression). Interestingly, RA DCs highly express pro-inflammatory transcription factor NF-κB in correlation with the disease activity, and in response to local inflammatory mediators such as damage-associated molecular patterns (DAMPs) and TNF-α (77). The abnormally active DCs suggest the failure of immune control mechanisms, possibly including joint MSCs.

In RA, fibroblast-like synoviocytes or MSCs have been shown to function as antigen-presenting cells leading to T-lymphocyte activation and proliferation (78) when thrombospondin-1 expressed by these synoviocytes are engaged with CD47 on T-lymphocyte surface (79). Another mechanism that could explain how MSCs could act as DC-like cells is TLR activation directly involved in RA pathogenesis (80). TLR2 and 4 are highly expressed in the synovium (81) with the most abundant types of ligands for TLR2 and TLR4 including HSP22 and Biglycan (82, 83). A study has shown that RA-induced cytokines IL-12 and IL-18 together with IFN-γ cause an upregulation of TLR4 on synovial MSCs and consequently trigger the expression of pro-inflammatory cytokines IL-6 and TNF-α (81). The TLR-related pro-inflammatory functions of MSCs demonstrate MSC changes in RA, but it would be still valuable to investigate how DCs display their functions when co-cultured with synovial MSCs in RA milieu.

Plasmacytoid dendritic cells (pDCs) are the primary producers of IFN-α (84). In SLE, pDCs induce the differentiation of immature B-lymphocytes to plasma cells but not B-reg cells with induction of IFN-α secretion (85). In parallel, high IFN-α in SLE marked by increased plasmablasts and CD314 (86, 87) has been linked to defective T-reg cell function that promotes differentiation of CD4+ T effector cells (88). In contrast to hematopoietic cells, IFN-α seems to have less effect on MSCs. The gene expression levels of differentiation markers of native MSCs were found unaffected by IFN-α (89). However, when MSCs were treated with IFN-α within an *in vitro* culture condition, inhibition of cell proliferation was reported (89, 90). Additionally, the osteogenic differentiation potential of culture expanded MSCs was found to be suppressed by exposure to IFN-α (91). Considering IFN-α is one of the major players in SLE milieu, it remains to be established if this cytokine could affect immunomodulatory functions of MSCs at a functional level. Altogether, MSCs and DCs can behave differentially in normal condition, however these cells share some mechanisms related to pro-inflammatory cytokine release and TLR activation in autoimmune diseases such as RA and SLE.

### Macrophages and MSCs

Macrophages are a source of bone morphogenetic proteins (BMPs) and Oncostatin M, which induce the proliferation and osteogenic differentiation of MSCs (92) and conversely, MSCs regulate macrophage function. Bone marrow MSCs can suppress monocyte differentiation into osteoclast via osteoprotegerin production (OPG) (93). Also, MSCs can drive macrophage polarization from their pro-inflammatory to anti-inflammatory types through the production of IDO, COX-2/PGE2, and TSG-6 (94). By a PGE2-dependent mechanism and IDO involvement, human MSCs attenuate pro-inflammatory function and enhance the anti-inflammatory role of macrophages that have increased phagocytic capacity and high production of IL-10 and TGF-β (95). Human MSCs also affect macrophages' functions to overproduce IL-10 and reduce IL-12 and TNF-α expression levels (96). Collectively, MSCs can induce the anti-inflammatory effect of macrophages.

Inflammatory joint resident macrophages in RA are stimulated by Th1 cells via IFN-γ to produce IL-1β and TNF-α acquiring pro-inflammatory phenotype and inducing a hyperimmune response in RA inflamed joint synovium (97). In parallel, MSCs within such an inflammatory microenvironment could also induce macrophage pro-inflammatory phenotype via TLR2 and TLR4 induction (4). Furthermore, RA-MSCs could have innate immune abilities with a production of GM-CSF that is a major macrophage activating factor (98). These data indicate the cumulative effect of MSCs and macrophages in the progression of arthritis.

In addition to their role in chronic inflammation, macrophages are involved in bone and cartilage repair. Macrophages can induce the expression of RANKL by MSCs promoting the osteoclast formation (99). Similarly, MSCs were shown to induce the osteoclastogenesis from BM hematopoietic stem cells although in a different mechanism via involvement of IL-6 (100). Furthermore, macrophages produce matrix metalloproteinases (MMPs) that aid to degrade the cartilage matrix (101). Additionally, macrophages produce TNF-α within inflammatory milieu reduce osteoblasts and promote osteoclasts (102, 103). TNF-α stimulates the production of M-CSF by MSCs, which, in turn, induces the differentiation of osteoclast progenitors (104). With regards to cartilage, TNF-α induces the death of mature chondrocytes and the growth of bone cells assisting the conversion from cartilage into bone (105). TNF-α also increases MMPs that could mediate cartilage degradation and angiopoietin production helping osteochondral angiogenesis (106). The bone and cartilage changes mediated by inflammatory cytokines could explain how MSCs within the inflammatory arthritis milieu cannot repair or overcome joint tissue damage. All these data demonstrate how chronic inflammation can affect immune cells and MSCs leading to joint tissue damage. While macrophages have a pro-inflammation effect and destructive role in RA joints' bone and cartilage, MSCs may be promoting these altered functions.

### T-Lymphocytes and MSCs

T-lymphocytes are central players in orchestrating inflammation in RA and SLE. Joint resident lymphocytes in health or disease are in contact with joint resident MSCs and thus exhibit complex interactions. The effect of T-lymphocytes on MSCs pervasively impacts on all MSC functions. In addition to licensing effect, T-lymphocyte-derived cytokines IFN-γ and TNF-α can upregulate MSC migration (107), but block the MSC differentiation capacity (108).

Compared to Th1 cytokines, Th17 cells were reported as an osteoclastogenic helper T-lymphocyte subset also contributing to bone damage in arthritis (109). Furthermore, IL-17 can suppress the chondrogenic differentiation of MSCs via suppression of a key chondrogenesis transcriptional factor, SRY-box 9 (SOX9) and its activator cAMP-dependent protein kinase (PKA) (110). Innate lymphocytes cells (ILC3s) and Th17 cells commonly produce IL-17, but ILC3s also produce IL-22 and M-CSF cytokines (111). We have demonstrated that IL-22 could stimulate the proliferation and osteogenic differentiation of IFN-γ/TNF-α-licensed MSCs (6) indicating the complexity of how inflammatory milieu can affect the regenerative capacity of MSCs.

While T-lymphocytes affect MSC function, MSCs can in turn, affect T lymphocytes. The culture expanded synovial MSCs extracted from healthy subjects can inhibit T lymphocyte proliferation (112). Also, these MSCs can maintain the percentage of T-reg cells when in co-culture with T-reg enriched lymphocytes (113) further supporting the role of MSCs in maintaining immune tolerance. Some studies indicated that MSCs from RA patients could preserve T lymphocytes immunosuppression capacity *in vitro*. The co-culture of PBMCs with BM-MSCs, both from RA patients or healthy controls, resulted in decrease the production of TNF-α, IL-17, IL-6, IL-2, IFN-γ, and IL-9 by all T-lymphocyte subsets (naive, effector, and memory T-lymphocytes). Also, these MSCs induced the gene expression levels of anti-inflammatory cytokines IL-10 and TGF-β by CD4^+^ and CD8^+^ T-lymphocytes (114). Another *in vitro* study showed that human RA synovial MSCs, in a fashion comparable with donor-matched BM-MSCs, suppressed T-cell response in a mixed lymphocyte reaction with a similar expression level of IDO (112). In contrast, another study showed that RA MSCs had an impaired function in inhibiting Th17 cells (115). Instead, RA fibroblast-like synoviocytes stimulated T-cells following interaction between CXCR4 on T-lymphocytes and SDF-1 on synoviocytes (116). The variability in the capacity of RA MSCs to suppress T-lymphocytes suggests that the altered immunosuppressive mechanism of these MSCs could probably be correlated to disease severity or donor variability.

Human MSCs produce HLA-G5 a non-classical MHC class I molecule, which has been linked to inhibition of the reactivity and cytolytic function of alloreactive T-lymphocytes (117). HLA-G5 was also found to be involved in the suppression of T-lymphocyte proliferation as well as promoting the differentiation shift toward T-reg cells (117). In SLE, the link between high levels of IL-10 and HLA-G and chronic pro-inflammatory status is not clear. The overall MSC and T-lymphocyte interactions seem to be complicated to explain how both cell types collaboratively promote chronic inflammation in autoimmune joint diseases. However, these interactions explain clearly how the regenerative capacity of MSCs could be limited in this inflammatory milieu.

### B-Lymphocytes and MSCs

A supportive role for joint stromal and B-cell function in RA is a long-recognized phenomenon (86). Normal human MSCs can inhibit the proliferation and functions of B-lymphocytes (4). In contrast, RA fibroblast-like synoviocytes could promote B-lymphocyte migration, via SDF-1 and vascular cell adhesion molecule-1 (VCAM-1) dependent mechanisms (118, 119). Additionally, RA synovial stromal cell line could promote the survival and functions of B-lymphocytes via an increase in the expression of BCL-XL via a CD49/CD29-CD106-dependent mechanism (120). Also, RA synovial fibroblasts could promote B-lymphocyte survival by upregulation of IL-15 receptor on the surface of B-lymphocytes (121). Furthermore, RA fibroblast-like synoviocytes induced immunoglobulin class switching in B-lymphocytes (122). These data together indicate the changed function of MSCs toward B-lymphocytes in RA favoring inflammation.

BM-MSCs from SLE patients showed less immunosuppressive effects on B-lymphocytes compared to control MSCs. The effect of these SLE-MSCs on the proliferation and production of autoimmune antibodies was mediated via CCL2 (123). While BM-MSCs in SLE have defective immunosuppressive potential, these MSCs stimulated the growth and maturation of B lymphocytes (124). Furthermore, high expression of olfactory 1/early B-lymphocyte factor-associated zinc-finger (OAZ) gene that is linked to cell cycle control was detected for BM-MSCs from SLE patients. Interestingly, the down expression of this gene was associated with increased ability of MSCs to inhibit B-lymphocyte functions of autoantibody production (125). Although less characterized, MSCs seem to promote B-lymphocyte response in SLE in common with RA.

Collectively, MSCs seemed to be reprogrammed to support the pro-inflammatory functions of B-lymphocytes rather than immune response regulation in both RA and SLE. At the present, we would summarize the literature as suggesting that the multiple impacts of MSCs on the immune system outweigh any potential detrimental impacts on autoantibody production. Further investigations into the mechanisms underlying these changes would help to understand autoimmune pathogenicity and to optimize MSC-based therapies as discussed in the next sections.

## Therapeutic Use of MSCs in RA and SLE

### Animal Models of Using MSCs for Experimental RA and SLE

Several experimental models confirmed the potential effectiveness of MSCs for RA and SLE treatment via multiple anti-inflammatory mechanisms. Murine bone marrow MSCs have been shown to impair TLR-4 induced activation of DCs, thus reducing the DC migration to lymph nodes and excretion of antigen presentation (74). Additionally, UC-MSCs were effective for the treatment of RA, as shown in collagen-induced arthritis (CIA) mouse model. These MSCs reduced synovitis and articular destruction with declined levels of TNF-α and IL-1β in the serum and joints (126). Human AD-MSCs have been also effective in increasing T-reg cells and IL-10 levels and reducing the serum levels of TNF-α and anti-collagen type II in CIA model (127).

Similar to RA, murine SLE studies showed that the application of therapeutic human BM-MSCs showed a decrease in serum levels of anti-dsDNA antibodies and renal complement C3 expression (128, 129). Furthermore, allogeneic murine MSCs were effective in SLE mice compared with cyclophosphamide treatment (130). In another SLE experimental model, allogeneic MSCs enhanced the anti-inflammatory effects of immunosuppressant drugs with less adverse effects of these medications (131). In another study, allogeneic MSCs have reduced renal immunoglobulin G (IgG) deposition in SLE mice (132). Recently, a novel mechanism of how therapeutic MSCs can suppress chronic inflammation in SLE was described whereby transplantation of allogeneic UC-MSCs has been shown to increase the expression of CD1c^+^DCs by promoting their proliferation and anti-apoptosis effect (133). Although, MSCs were found effective in controlling immune responses in RA and SLE experimental models by effects on DCs and both T and B lymphocytes, the outcomes on disease severity and markers were variable, and the potential reasons are discussed below.

The source of MSCs is one of the common variables used in preclinical RA and SLE studies. *In vitro* studies showed that UC-MSCs, AD-MSCs and BM-MSCs similarly inhibit the maturation of DCs, T cell proliferation and activate T-reg cell induction (134–138). In contrast, the superiority of UC-MSCs was reported in hampering the cytokine release from LPS-stimulated macrophages and in the induction of T-reg cells compared to BM-MSCs (139, 140). Other studies showed that AD-MSCs are more potent in inhibiting T cell activation and proliferation than UC-MSCs and BM-MSCs (141, 142). In contrast to *in vitro* studies, fewer studies directly compared the efficacy of different MSC sources in RA models. Synovial fluid-MSCs effectively improved the severity of collagen-induced arthritis more than BM-MSCs (143). In the second study, BM-MSCs, AD-MSCs, and UC-MSCs treated arthritis with no significant difference between the three groups (144). The meta analysis data of RA experimental models showed that AD-MSCs and UC-MSCs had better therapeutic effects on experimental RA than BM-MSCs. Interestingly, clinical scores were similar between UC-MSCs and AD-MSCs, but UC-MSCs demonstrated better outcomes using histological tissue scores (bone erosion, cartilage damage, and inflammation) (145). Reviewing SLE experimental models, different BM, AD or UC-MSCs were comparably effective as shown by the reduction of T effector cell number, increased T-reg cell number in the spleen and decreased antibody production and inflammatory cytokines in kidneys (146). Overall, the *in vitro* and preclinical data suggested a similarity of AD-MSC and UC-MSC potency probably followed by BM-MSCs to induce immunosuppression for RA and SLE experimental models.

While some studies showed no difference in immune compatibility of MSCs (147–149), other groups reported that allogeneic and syngeneic transplantation had no different effect on arthritis (150, 151). In contrast, using xenogeneic MSCs e.g., human-derived MSCs in RA experimental studies was more effective than the use of allogeneic or syngeneic murine MSCs (145). These data suggest that an appropriate level of immune incompatibility between donor MSCs and the host may benefit cell therapy.

The quantity of MSCs is another factor that could affect the therapeutic outcome. UC-MSCs was effective in improving mouse CIA in a dose-dependent manner in another study (152). Other two studies showed that the higher doses (2.5, 5 × 10^6^) of either BM-, UC-, or AD-MSCs were more effective than a lower dose (1 × 10^6^) for treating arthritis mice models (144, 153) indicating that adequate quantities of MSCs are essential for satisfactory therapeutic effect. In terms of the frequency of the doses, two doses were shown to be more effective than a single dose, and the therapy timing was more important than the number of BM-MSC doses (149). Other two studies reported that there was no difference between single or double doses of 1 × 10^6^ BM-MSCs (150), or between two and five injections of AD- or BM-MSCs (144).

Interestingly, the administration time of the required quantity of therapeutic MSCs was investigated. Injection of MSCs during the early phase of disease was associated with favorable outcomes in RA animal models, and the earlier the MSC treatment, the more efficient treatment (153–155). However, in severe arthritis model, early MSC dose was not adequate to reduce the disease severity over the long term (149, 156). Overall, it is possible that variability in disease severity or the inherent immunomodulatory ability of injected MSCs could influence the outcomes using adequate doses of MSCs.

Route of administration was another factor to be assessed in preclinical models, and the data showed that injection of MSCs prior to the onset of arthritis via either IV or intra-peritoneal (IP) routes was similarly effective and better than administration after the onset of the disease (153). Furthermore, local implantation of MSCs, particularly when loaded on scaffolds were more effective in improvement of arthritis scores compared to intra-articular (IA) or IP (157). Finally, the passage number of culture-expanded MSCs and culture conditions are other factors that might impact the immunomodulatory potential of MSCs and necessitate further investigation.

### Clinical Studies of Using MSCs for RA

The value of MSCs in autoimmune diseases or transplantation is mainly related to their basic immunosuppressive capacity and the bonus of a good safety profile (158). As mentioned, the immune system may activate inflammatory responses in MSCs; therefore, MSCs from non-inflamed environments may conversely modulate aberrant immune responses. Thus, the use of healthy MSCs is a promising tool for RA therapy particularly in the therapy of refractory disease (159).

There is a relative paucity of randomized controlled trials (RCT) of MSCs in RA and SLE and beyond that also a general lack of trials in other immune diseases settings (37, 160). Nevertheless, RCTs in the transplantation setting have suggested efficacy and safety of MSCs (158, 161). A systematic literature review and meta-analysis of MSC trials showed that only a minority were undertaken for an inflammatory disease where numbers were generally small, but safety was good (158, 161). It can be surmised that the promising laboratory mechanistic data on MSC immunomodulation and the supportive preclinical data, but there is a paucity of high-quality RCTs in RA and SLE.

The MSC-based therapy usually involves culture expansion of either autologous or allogeneic MSCs, and these cells are infused intravenously at an average of 1–2 million MSCs per kg of body weight (162). Several studies have investigated the use of (UC-MSCs in RA clinical trials (Table 1). Clinical studies using MSCs for treating refractory RA have considerably increased since 2011 with UC-MSCs used in nearly half of these studies. Therefore, it is not surprising that the therapeutic MSCs are 78% allogenic and 22% autologous. Additionally, both one dose and multiple doses are popular. Two early pilot studies have proved safety for MSCs in RA therapy. Ra et al. used autologous AD-MSC therapy for four RA patients, among other autoimmune diseases. These MSCs were infused intravenously in a single, double, or quadruple dose. The patients were monitored for 13 months, with all of them showing no adverse effect and improvement of Visual Analog Scale (VAS) and Korean Western Ontario McMaster (KWOMAC) scores (176). In another small pilot study, four refractory RA patients were treated with allogeneic BM-MSCs or UC-MSCs. These patients were given a single IV infusion of one million MSCs/kg of body weight. MSC-based therapy proved no adverse effects, and the therapy seemed less effective. After 24 months, three out of four patients had a moderate response measured using European league against rheumatism (EULAR) parameters, erythrocyte sedimentation rate (ESR), C reactive protein (CRP), disease activity score (DAS) 28, and VAS score (165).

**Table 1:** Clinical trials for RA therapy using MSCs.

**Phase**	**MSC source**	**Patients**	**Outcome**	**References**
Phase I/II	UC allogeneic	64 patients	Three years follow-up: no serious adverse effects. Remission measured by ACR, DAS-28, ESR, and HAQ. Low levels of CRP, RF, anti-CCP antibodies, TNF-α and IL-6. Increase of blood T-reg cells.	(163)
Phase I/II	UC allogeneic	172 patients	The 8-month follow-up; no serious adverse effects Low levels of CRP, RF, anti-CCP antibodies, TNF-α and IL-6. Increase of blood T-reg cells.	(164)
Phase I	UC allogeneic	4 patients	3 patients had lower ESR, DAS-28, pain VAS score at 1 and 6 months. 2 patients had a EULAR moderate response at 6 months but a relapse at 7 and 23 months, respectively. No one had achieved the DAS-28-defined remission. No serious adverse events were reported.	(165)
Phase I/II randomized	UC allogeneic	105 patients refractory	Safety and good response indicated by initial IFN-γ induction then increase of IL-10 and T-reg cells/TH17 ratio.	(166)
Phase I	UC allogeneic	9 patients refractory	No major toxicity up to 4 weeks after the infusion. Reduction in serum erythrocyte sedimentation rate and DAS28 score. Reduced levels of IL-1β, IL-6, IL-8, and TNF-α.	(167)
Phase I/II	UC allogeneic	63 patients refractory	Efficacy and ACR20 response rates in 53.3% patients with MSC and in 93.3% patients with MSC combined with IFN-γ at 3-month follow-up. No new or unexpected safety issues were encountered in 1-year follow-up.	(168)
Phase 1	UC allogeneic	9 patients refractory	Safety, a decline in the DAS28-ESR, HAQ, and VAS scores as well as blood levels of ESR and CRP, IL-1β, IL-6, IL-8, and TNF-α.	(167)
Phase I/II non-randomized	UC allogeneic	64 patients refractory	Safe after 1 and 3 years. The ESR, CRP, RF were lower than that of pre-treatment. Decrease in HAQ and DAS28 scores.	(163)
Phase I	BM autologous	9 patients refractory	Increase T-reg cells. Decreasing trend in Th17. Decrease DAS28 and VAS scores.	(169)
Phase I	BM autologous	13 patients refractory	Increase gene expression of FOXP3 at month 12. Increasing in PBMC culture supernatant levels of IL-10 and TGF-β1.	(170)
Phase I/II randomized	BM autologous	30 patients	No adverse effects. Improvement in WOMAC, VAS score, but not beyond 12 months.	(171)
Phase I/II	BM autologous	20 patients Early RA	Ongoing NCT03186417^a^	
Phase I/II randomized	Adipose allogenic	53 patients	Well-tolerated therapy. ACR20 was 20–45% at 1-month and 0–25% at 3-month follow-up.	(172)
Phase II	BM-MSCs autologous	48 patients	There were positive clinical outcomes as observed using ACR, PGA, and HAQ.	(173)
Phase I	BM-MSCs autologous	13 refractory patients	A decrease in the blood levels of CD19+ B cells with a decreased expression of BR3, TACI, and BCMA receptors and blood BAFF and APRIL levels 12 months after the MSC infusion.	(174)
Phase I	BM-MSCs autologous	13 refractory patients	The blood levels of CXCL8, CXCL12, and CXCL13 were significantly decreased 6 months after MSCs transplantation but returned to pre-treatment levels after 12 months.	(175)

a *ClinicalTrials.gov. Available online at: http://clinicaltrials.gov/*.

*BM, Bone marrow; UC, Umbilical; DAS28, 28-joint disease activity score; HAQ, Health index; EULAR, European League Against Rheumatism; FOXP3, forkhead box P3; WOMAC, Western Ontario and McMaster Universities Arthritis Index; VAS, visual analog scale; ACR20, American College of Rheumatology 20 responses; PGA, Patient global assessment*.

Wang et al. have conducted two studies; one phase I/II clinical trial treating 172 RA patients with up to 8-month follow-up (164) and another study with 64 patients were followed up to 3 years for long term results (163). In both studies, patients were treated with a single dose of systematically injected allogeneic UC-MSCs (4 × 10^7^ cells/patient) combined with low-dose DMARD treatments (leflunomide, hydroxychloroquine sulfate, or methotrexate). The outcomes were no serious adverse effects and a remission measured by the American College of Rheumatology improvement criteria (ACR), the DAS-28, ESR, and the Health Assessment Questionnaire (HAQ). Additionally, low levels of CRP, rheumatoid factor (RF), and anti-cyclic citrullinated peptide (anti-CCP) antibodies, TNF-α and IL-6 cytokines were reported. These clinical results, in addition to the increase in the percentage of blood T-reg cells were reported in both studies (163, 164).

A small study was conducted on nine RA patients who were not treated with any biologic compound but received IV allogeneic UC-MSCs. In this phase I clinical trial, the MSC doses were 2.5 × 10^7^, 5 × 10^7^, or 1 × 10^8^ UC-MSCs per subject (167). The safety, the decline in the DAS28, HAQ, and VAS scores, as well as blood levels of ESR, CRP, IL-1β, IL-6, IL-8, and TNF-α, particularly with the higher dose of MSCs were noted.

Despite the promising effects of UC-MSCs in the above clinical studies, other reports indicated less effectiveness of using allogenic UC-MSCs even with a higher dose. A phase I clinical study treated 53 refractory RA patients with a single IV dose of 1 × 10^6^ allogeneic UC-MSCs/kg of body weight. The results of up to 12 months follow-up confirmed the clinical safety of this MSC therapy (166). However, the clinical outcomes were variable with 54% of the patients achieved a good or moderate response, but 46% of the patients had no clinical response as measured by CRP, ESR, HAQ and DAS28 scores. Also, 8% of responders experienced a relapse by 24 weeks. With regards to laboratory findings, responders had increased blood levels of albumin, hemoglobin, T-reg cells and IL-10, but a decrease in anti-inflammatory mediators anti-CCP, IL-6, TNF-α, and Th17 cells. Interestingly, there was a positive association between high serum IFN-γ levels and reduced DAS28 score. In agreement, a recently published clinical study for 63 refractory RA patients showed that 1 × 10^6^ IU of recombinant IFN-γ that injected intramuscularly increased the effectiveness of simultaneous delivered UC-MSCs (intravenous 1 × 10^6^ MSCs/kg of body weight) by nearly 2-fold (168). Together these data further confirmed the perception that MSC immunosuppressive capacity is not constitutive, but dependent on a process of cytokine-related licensing that is acquired within inflammatory milieu and could be different between patients.

Another source of therapeutic MSCs is bone marrow with several studies that have been completed or ongoing (Table 1). In a multicenter randomized double-blind placebo-controlled sequential dose-escalation phase II study, allogeneic BM MSCs with high differentiation capacity expressing STRO-1 or STRO-3 markers were used (173). An infusion of 1 × 10^6^ or 2 × 10^6^ MPCs/kg of body weight were used in 48 patients in combination with DMARDs. There were positive clinical outcomes as observed using ACR, patient global assessment (PGA), and HAQ particularly with the higher dose. Furthermore, a pilot study involved nine refractory RA patients who were IV-infused with one dose of autologous BM-MSCs (1 to 2 × 10^6^ cells/kg of body weight) combined with conventional therapy. Twelve-month follow-up showed no adverse effect, a significant decrease in DAS28-ESR, VAS scores and ESR, but no changes in blood inflammatory markers (169). Systemic injection of autologous BM-MSCs into refractory RA patients with 12 month-follow up demonstrated that the gene expression of forkhead box P3 (FOXP3) in peripheral blood mononuclear cells (PBMCs) was significantly induced (170). Furthermore, high levels of IL-10 and transforming growth factor-beta 1 (TGF-β1) were noted in the culture supernatant of PBMCs over the time course of treatment (170). Autologous BM-MSCs were also used in another study with promising effects, but up to 12 months follow up (171).

The first randomized multicenter clinical study using allogeneic AD-MSCs for RA was a double-blind placebo-controlled dose-escalation phase Ib/IIa trial (172). Fifty-three refractory RA patients were divided into three groups, having intravenous infused MSC doses of 1, 2, or 4 × 10^6^ cells/kg of body weight. After a 6-month follow-up, a transient fever was noted, but a satisfactory response was observed using the EULAR criteria, DAS28-ESR, and CRP. No MSC dose-response was noted, probably because of the small number of RA patients in each group. With regards to inflammation markers, no significant changes in circulating T cell populations were observed. Interestingly, there was an indication of MSC immunogenicity in 19% of RA patients, but without apparent clinical significance (172).

A recent clinical study for 13 RA patients suggested that multiple doses of BM-MSCs or a higher dose of MSCs may be required to maintain immunosuppressive status (174). Also, this study has reported for the first time a decrease in the blood levels of CD19+ B cells with a decreased expression of BLyS receptor 3 (BR3), transmembrane activator and CAML interactor (TACI), and B-cell maturation antigen (BCMA) receptors and blood BAFF and APRIL levels 12 months after the MSC infusion. These results indicate that MSC therapy may decrease B cell proliferation. The same research group showed that blood levels of CXCL8, CXCL12, and CXCL13 were significantly decreased 6 months after BM-MSCs transplantation but returned to pre-treatment levels after 12 months (175).

Altogether, several clinical trials have offered support for the safety of MSCs particularly for resistant RA (165, 166, 168, 170–172). The variable outcomes regarding remission could be due to MSC type, dose, and frequency as well as donor factors such as age, health condition and immune status. Higher and multiples doses and availability of inflammatory licensing milieu for MSCs seem to favor better outcomes. Nevertheless, more randomized controlled trials are needed to establish efficacy. The source of therapeutic MSCs is an important point to consider for treating autoimmune arthritis particularly with tissue damages/lesions. Considering research findings that are indicating that autologous RA MSCs might not act efficiently as immunosuppressive cells, it is not surprising that allogenic MSCs are frequently used in RA patient studies. Most of the clinical trials have involved the systematic infusion of allogeneic MSCs from BM, AD, and UC (177). Furthermore, the therapeutic effect of injected allogeneic MSCs could be diminished probably due to activated immune mechanisms (178). More research is needed to compare how activated immune response and inflammatory milieu can affect the activities of infused allogeneic and autologous MSCs.

### Clinical Studies of Using MSCs for SLE

The therapeutic management of SLE has historically focused on the use of intensive immunosuppressive agents such as steroids, methylprednisolone, cyclophosphamide and mycophenolate mofetil and latterly drugs that target B-cell and autoantibody axis (179). However, potentially serious side effects related to immune suppression e.g., infection and myelosuppression, as well as organ toxicity can arise (180, 181). Furthermore, some patients are resistant to these standard therapies (182). Consequently, there is a need to develop new approaches for SLE treatment.

Several clinical trials of MSCs in SLE are either completed or ongoing (Table 2). Application of therapeutic MSCs is a fairly new tool for SLE patients, but reported as safe with claims of effectiveness in refractory disease patients (184–186, 188, 190). One study tested the effect of intravenous infusion of UC-MSCs at 1 × 10^6^/kg body weight to treat nine refractory SLE patients. No adverse events such as headache, nausea or vomiting were observed. There was no change in the blood picture, organ functions or tumorigenic marker levels (188). In another study, UC-MSCs were used for several autoimmune diseases, including SLE. The data showed 5-year and 8-year survival rates were 90.4 and 88.9%, respectively. The incidence rate of infections was 29.5, and 1.2% of patients experienced malignancies. Deaths rate was 0.2% mainly due to disease relapse and complications associated with the underlying disease (185). In another study, three patients were given 90 × 10^6^ allogenic BM-MSCs and were followed up for 9 months. The results showed improvement as monitored by the SLE disease activity index (SLEDAI) and other markers such as proteinuria, lymphocyte and monocyte antigens antibodies (184). In contrast, 13 SLE patients were also treated with allogenic BM-MSCs with no adverse effect, but 2 out of 13 patients had disease replace (186). In a randomized study, 2 out of 12 patients treated with UC-MSCs had pneumonia while one patient in the control group (6 patients) had a stroke, and another had ascites (190).

**Table 2:** Clinical trials for SLE therapy using MSCs.

**Phase**	**MSC source**	**Patients**	**Outcomes**	**References**
Case study	BM autologous	2 patients	MSCs induced T-reg cells. No effect on disease at 14-week follow-up. One patient had a renal flare.	(183)
Phase II	BM allogenic	3 patients, high activity	SLEDAI scores: substantial remissions for 2 patients and partial for the third.	(184)
Phase I/II	BM allogenic	15 refractory patients	Decrease in Anti-dsDNA levels & the SLEDAI score at 12-month follow-up. 2 patients had a renal relapse. No serious adverse events were reported.	(185)
Phase I/II	BM/UC allogeneic	404 SLE and other autoimmune patients	The 5- and 8-year survival; 90.4 and 88.9%, respectively. Rate of infections was 29.5% and serious infections was 12.9% 1.2% patients experienced malignancies. MSC transplantation-related mortality was 0.2%.	(186)
Phase II	BM/UC allogeneic	81 refractory patients	60.5% remission rate. Improvement of GFR, BILAG and SLEDAI scores during 12-month visit by MSCT. 22.4% had experienced renal flare by 12-month follow-up Total disease activity evaluated by Systemic Lupus	(187)
Phase II	UC allogeneic	81 refractory patients	Good safety profile of MSCs in SLE patients. Normal Liver and heart function. No change in peripheral blood cell counts. No rise of serum tumor markers.	(188)
Phase II	UC or BM allogenic	87 refractory patients	Half of the patients entered clinical remission at 4 years. No adverse event was observed.	(189)
Phase II	UC allogeneic	18 patients	MSCs had no apparent additional effect. One patient had leukopenia, pneumonia and another died of severe pneumonia.	(190)
Phase II	UC allogenic	16 patients, refractory or had life-threatening visceral involvement	Increase in peripheral T-reg cells, balance between Th1 and Th2 cytokines. Significant reduction in disease activity in all patients (SLEDAI score, levels of serum ANA, anti-dsDNA antibody, serum albumin, and complement C3, and renal function. No recurrence, no treatment-related deaths	(191)
Phase I/II	UC allogenic	40 patients, active SLE	SLEDAI and BILAG scores were significantly improved. Decrease of serum antinuclear and anti-double-stranded DNA antibodies 0.12% disease relapse after 6 months.	(192)
Phase II	UC allogenic	81 patients	Ongoing, NCT02633163^a^	
Phase I	UC allogenic	7 patients	Not reported yet, NCT03174587^a^	
Phase I	UC allogenic	6 patients	Not reported yet, NCT03171194^a^	
Phase I/II	Olfactory mucosa	10 patients	Ongoing, NCT04184258^a^	
Phase I	UC allogenic	10 patients	Ongoing, NCT03219801^a^	
Phase I	Amniotic fluid	16 patients	Not reported yet, NCT04318600^a^	

a *ClinicalTrials.gov. Available online at: http://clinicaltrials.gov/*.

*BM, Bone marrow; UC, Umbilical; BILAG, British Isles Lupus Assessment Group; SLEDAI, The Systemic Lupus Erythematosus Disease Activity Index 2000*.

Several studies demonstrated laboratory signs of SLE disease activity improvements. Using MSC therapy in either case studies or RCTs showed a reduction in SLE laboratory disease activity markers such as proteinuria (187, 190, 193). With regards to clinical remission, the results were variable. When 2 × 10^8^ UC-MSCs were infused intravenously in 12 SLE patients, remission occurred in 75% of these patients compared to 83% remission in who had placebo (190). In a multi-center clinical study, two doses of intravenously infused UC-MSCs were well-tolerated with no adverse effects (192). Additionally, SLEDAI and BILAG scores for renal, hematopoietic and cutaneous systems were significantly improved. Blood levels of antinuclear antibody and anti-double-stranded DNA antibodies also decreased significantly at 3-month follow-up. There was 12% disease relapse detected after 6 months of follow-up (192). A 4-year study showed that half of 87 treatment-resistant SLE patients entered clinical remission at 4-year follow-up after allogenic BM- or UC-MSCs were infused intravenously (189). In this study, the popular dose (1 × 10^6^ MSCs/kg of body weight) was used, but the overall rate of relapse was relatively high (189). In another study, UC-MSC infusion induced 100% disease remission for 16 resistant SLE patients after 28 months (191). Significant remission was reported according to the SLEDAI score, blood levels of anti-dsDNA antibody, albumin, Complement-3, and renal function. Additionally, an increase in T-reg cells and normalized Th1- and Th2-related cytokines were observed (191).

In a case study, autologous BM-MSC transplantation had no effect on SLE disease activity in 14 weeks of follow-up despite increasing T-reg cell counts (183). The transplantation of autologous MSCs could probably fail to inhibit the pathogenic immune reactions in SLE patients due to defective immunomodulatory functions (194). Both intrinsic and microenvironmental mechanisms seemed to be related to the defective functions of SLE MSCs. Therefore, improving the functions of autologous MSCs can be an alternative therapeutic approach. Collectively, most clinical studies up to date showed that MSC therapy for SLE had good but variable efficacy with 50–100% remission rate. Like RA clinical trials, the variation in the effectiveness of these MSC therapies could be related to the dose and type of MSCs, the clinical status of patients prior to therapy and the concurrent use of other immunosuppressive regimes. Most of the SLE clinical studies included a single dose of MSCS and showing variable outcomes. Therefore, larger studies are needed to validate the efficient dose and need for biological/inflammatory mediators.

### Non-cellular and Apoptotic MSC-Based Therapy for RA and SLE

Extracellular vesicles (EVs), a part of MSC secretome, have recently attracted considerable attention as new therapeutic vehicles for treating RA and other immune-related diseases (195). The EVs are small vesicles excreted by parent cells, which carry nucleic acids, mitochondria and proteins within a lipid-bilayer membrane, and they can fuse with recipient cells, thus enabling direct cell-to-cell communication. MSC-derived EVs contain a large repertoire of miRNAs that can effectively regulate recipient's cell transcription toward inflammation reduction; furthermore, this MSC-EV cargo can be modified by MSC pre-conditioning. For example, the anti-inflammatory properties of MSC-EVs can be enhanced by MSC stimulation with TNF-α combined with IFN-γ (196). EVs derived from human MSCs containing miRNA-124a could help to reduce RA synovial hyperplasia as shown to inhibit the proliferation and migration of fibroblast-like synoviocyte cell line (197). In a porcine model of synovitis, BM MSC-EVs delivered intra-articularly, significantly reduced leukocyte counts and TNF levels in the synovial fluid (198). Thus, MSC-derived exosomes could be a new strategy for the treatment of RA.

The potential use of MSC-EVs for the treatment of SLE is also being explored. In contrast to MSCs themselves, EVs are easier to characterize and store, and being acellular entities, they carry no concerns associated with MSC tumorigenicity or the risks of immune responses (in case of allogeneic cells). The success of preclinical testing of MSC-EVs for graft-vs.-host-disease or a mouse model of acute kidney injury indicates that the use of MSC-EVs for SLE treatment may hold future promise. However, there are currently only a few preclinical studies describing the use of MSC-EVs in SLE animal models (199, 200) and none so far in human patients.

Altogether it is safe to use MSCs or their EVs in RA treatment with some encouraging response noted, more work is needed to determine optimal MSC sources, concentrations and routes of administration. Future work should also consider the choice of the intervention therapy time in relation to inflammation status and it is a challenge that needs to be addressed by having more molecular biology studies. Similarly, there is a perception that allogeneic MSCs are a potential therapeutic tool for SLE unlike autologous MSCs, which have a defective immunomodulatory function and poorly proliferative. Even with possible immunological rejection, allogeneic MSCs are the usual vehicle for putative MSC based immunomodulatory therapies. Targeting autologous MSCs for improving their function could be another option. Future studies should aim to investigate the mechanism of the functional defect of SLE MSCs. Such data will improve our understanding of the pathogenesis of SLE and could introduce new methods modifying cell therapy using autologous MSCs for SLE and other autoimmune diseases.

In addition to secretory products from viable MSCs, the apoptotic MSCs have been shown to induce immunosuppression effects. Galleu et al. showed that MSCs used to treat graft-vs.-host disease (GvHD) undergo apoptosis in a perforin-dependent mechanism by recipient cytotoxic cells, and this process is required for MSC-mediated immunosuppression (201). Interestingly, the group reported that the response of patients with GvHD to therapeutic MSCs is positively correlated with high cytotoxic activity against infused MSCs. Furthermore, it has been shown that apoptotic MSCs, which are engulfed by recipient macrophages could induce the production of IDO, thus mediating immunosuppression (201). Therefore, the infusion of apoptotic MSCs generated *ex vivo* could be an alternative concept for new MSC-based therapies of autoimmune diseases such as RA and SLE.

## Discussion

**Figure 1:**
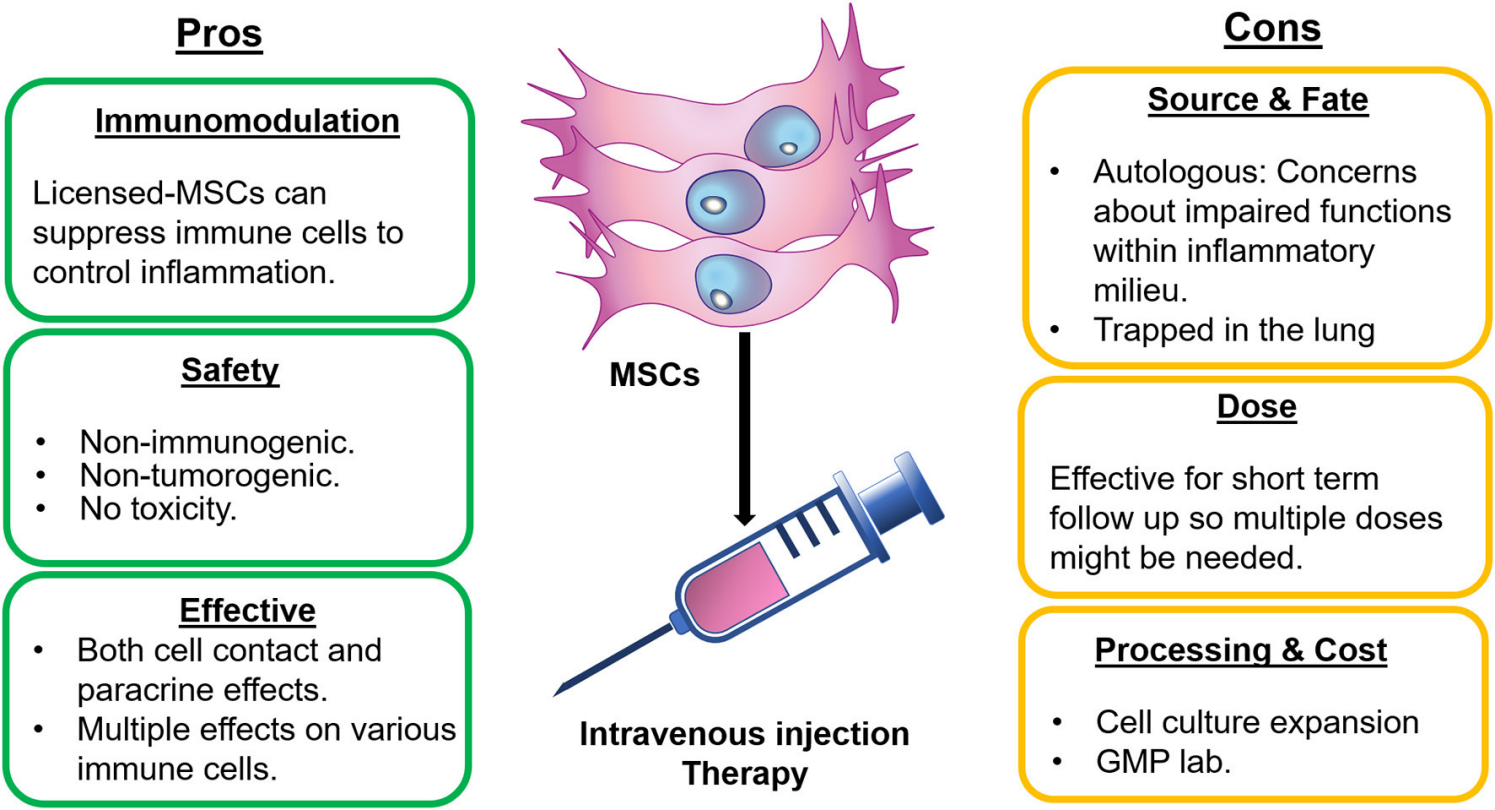
Pros and Cons of using MSCs for RA and SLE therapies. Therapy of RA and SLE using MSCs have some advantages including the immunosuppression capacity. These cells are safe to use and having simultaneous effects on multiple immune cells and exhibition of both contact and non-contact activities. In contrast, autologous MSCs might not be effective or only effective for short term due to inflammatory milieu. Also, intravenously injected MSCs could be trapped in lungs. Additionally, this therapy usually necessitates GMP lab for expansion of MSCs in culture.

**Figure 2:**
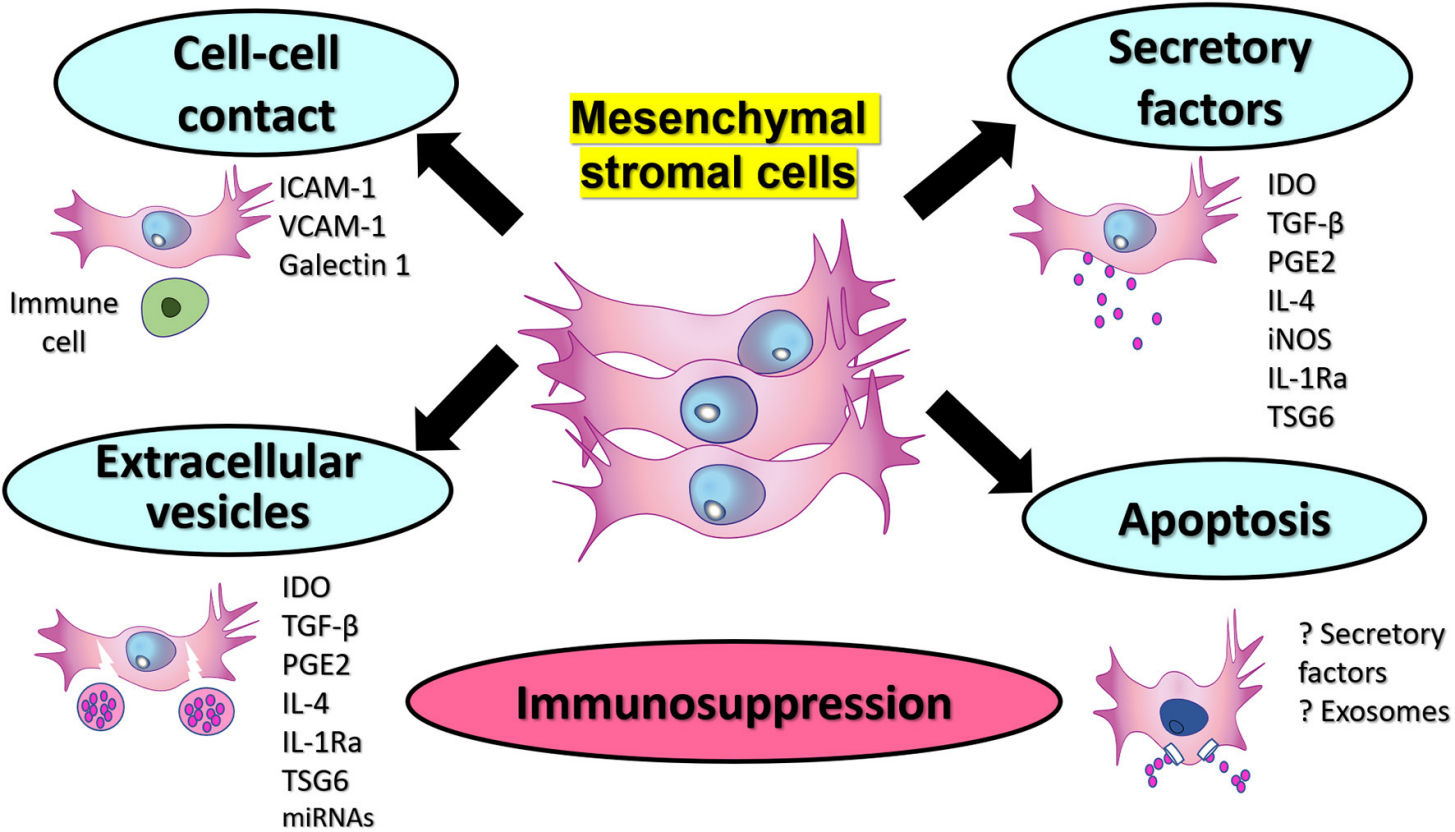
Potential mechanisms of immunosuppression by therapeutic MSCs. Therapeutic MSCs could display immunosuppression via production of extracellular soluble factors. Cell to cell interactions between MSCs and immune cells can be mediated via surface molecules such as ICAM-1, VCAM-1, and Gelactin-1. Additionally, extracellular vesicles containing immunosuppressive factors and other proteins and miRNAs constitute another mechanism that could be used therapeutic for autoimmune diseases. Finally, apoptotic MSCs that can be engulfed by macrophages, and this could probably help to mediate immunosuppression via soluble factors or extracellular vesicles or exosomes.

The use of MSCs as cell therapy for an autoimmune disorder such as RA or SLE is promising, still some aspects of treatment need consideration and further clinical testing and optimization is required (Figure 1). Typically, the immunosuppressive effect of licensed-MSCs is the rationale behind using them as a potential tool for treating autoimmune diseases. Uniquely, several mechanisms have been proposed for how MSCs can display immunosuppression (Figure 2). These mechanisms include the production of soluble factors, cell-cell interactions, extracellular vesicles, and recently described apoptosis-mediated immunosuppression. The best characteristic mechanism is secretory products followed by cell to cell mechanisms. Newly emerged EV and apoptotic MSCs are more recent though interesting mode of MSC immunosuppressive functions (36, 202). Nevertheless, combined actions of MSCs is likely but need further verification. Furthermore, the simultaneous effect of MSCs on multiple immune cells makes them suitable for use in refractory autoimmune arthritis. The safety of MSCs in human therapies has been well-demonstrated with no major toxicity. The possibility of excess immunosuppression and tumor transformation was noted in experimental models of arthritis treated with MSCs, although none was recorded in human studies (203). A milieu of chronic inflammation is associated with autoimmune diseases, and such a milieu could affect the functions of MSCs (7). Synovial MSCs in RA and SLE might directly participate in the disease progression. In active disease, these MSCs seem to have pro-inflammatory phenotype and they are not effective in controlling the exaggerated immune response. The functions of MSCs in SLE and RA could be changed due to multiple mechanisms that are known, however more studies are also needed to investigate the causes. It is important to assess the immunomodulatory functions of native MSCs to verify if the alterations of these functions are related to cellular changes, microenvironment or both. Interestingly, we have assessed by *in vitro* assay of T-cell proliferation how uncultured non-hematopoietic cells of cancellous bone can display immunosuppressive effects (33).

Different factors could affect the outcomes of MSC-based clinical trials for RA and SLE. As discussed above, the high doses, early timing and more frequency of MSCs seem to correlate positively with the therapeutic outcomes. Additionally, licensing by inflammatory milieu, particularly IFN-γ, is an important factor to consider, but this may increase regulatory issues substantially and delay or deter translational research in this area. The extraction method, accessibility and processing could be other factors affecting the choice of one type of MSCs rather than others (204). While several sources for MSCs have been utilized for clinical trials, UC-MSCs are the most frequent, followed by BM-MSCs and AD-MSCs. BM-MSCs are the best characterized MSCs and proven safe for use in the different regenerative and inflammatory application. With regards to accessibility, UC is a non-invasive source of MSCs, while harvesting BM-MSCs or AD-MSCs would require a surgical procedure, could sometimes be hard to access and cause complications (204). Additionally, MSC yield and functional potential are dependent on donor factors (e.g., age and co-morbidity) (89, 205). Several *in vitro*, preclinical and clinical studies remain inconclusive but suggest that the immunosuppressive function of UC-MSCs and AD-MSCs could be more potent than that of BM-MSCs (137, 139, 145, 206). However, the source/type of MSCs is not currently considered a factor affecting the clinical therapeutic outcomes, particularly with no clinical studies comparing the outcomes side-by-side between the different types of MSCs.

All clinical trials of using MSCs in RA or SLE employed culture-expanded MSCs as these expanded cells provide adequate quantity for multiple and high doses. However, these *ex vivo* manipulated cells would require the FDA's regulations for testing and approval of culture, expansion and treatment, which are essential to avoid any complication, e.g., infection (207). Although the processing of MSCs is a costly process with complicated steps for safety and quality control (208), this therapy still has great potential, particularly for patients with refractory diseases. The alternatives could be native or minimally manipulated MSCs. The main problem of these native uncultured MSCs is the limited numbers and variability due to donor age and health conditions (23, 209, 210). Therefore, some additional procedures were suggested to enrich the native sources of MSCs, such as BM concentration (187).

In addition to the quantity, several studies have aimed to improve the quality of therapeutic MSCs, particularly autologous cells using genetic modifications or biological stimulation. Genetically modified BM-MSCs to overexpress IL-37 showed *in vitro* suppression of splenocyte proliferation, decreasing cytokines IL-1β, TNF-α, IL-17, and IL-6 and autoantibodies leading to an improvement of SLE signs in a mouse model (211). BM-MSCs in SLE have higher expression of miR-663, which is correlated with SLE disease activity, and miR-663 inhibition was found to reduce the disease progression in SLE mouse model (194). Other examples include overexpression of miR-146a in MSCs that was associated with increased T-reg cells, FoxP3, TGFβ and IL-10 gene expression in CIA mice (212). Additionally, miR-320a inhibits the progression of RA and its induction in MSC exosomes or its target, CXCL9 helped to attenuate arthritis and bone destruction in CIA mouse model (213). It has also been shown that the transfection of IL-Ra gene in MSCs with long-term delivery via encapsulation in alginate-poly-L-lysine microcapsules can attenuate the inflammatory markers in CIA rat model (214). Other biological methods were proposed to augment autologous functions of MSCs. Endoplasmic Reticulum (ER)-stressed UC-MSCs further down-regulated peripheral CD4+CXCR5+ICOS+ T-lymphocytes associated with PGE2 and IL-6 increase in co-culture supernatant, suggesting that the immunosuppressive effect of MSCs could be enhanced by induction of ER stress (215). IL-4 has been shown as a potential therapeutic tool for autoimmune arthritis as its use in CIA mouse models was associated with an elevation of IL-13 levels and decreased IL-6 plasma concentrations (216). The mechanism of IL-4-linked immunosuppression is the ability to promote macrophage polarization into immunosuppressive cells via the suppression of Th1-mediated immune response as shown in proteoglycan-induced arthritis mice model (217). Similarly, IL-4 could increase MSC immunosuppressive effects by induction of T-reg cells as tested in a rat model of autoimmune colitis (218). While appear promising, genetic or other biological manipulations of RA and SLE MSCs can still carry considerable regulatory, ethical or safety concerns which may overweigh their therapeutic value.

The fate of infused MSCs is an interesting topic of cell-based therapeutic research. A model was proposed for a multistage process when MSCs enter the bloodstream as tethering and rolling, activation, arrest, diapedesis, and migration (219). Furthermore, preclinical studies demonstrated the presence of MSCs in inflamed tissues, and these MSCs able to induce different immunosuppressive, angiogenic, and anti-apoptotic effects (220, 221). Still, some challenges have been reported for intravenous infusion of MSCs. The systemic infusion of MSCs induces the expression of procoagulants like tissue factor (TF) on the surface of MSCs inducing instant blood-mediated inflammatory reaction (IBMIR) and complement activation. These reactions could induce cytotoxicity of infused MSCs (222, 223). Additionally, strong evidence showed that systematically injected MSCs are mostly trapped in the lungs, and few of these cells could circulate and reach the target site (224). Several methods have been suggested to overcome this issue by enhancing the migration of MSCs, modulating MSC surface markers or use of 3D cultures (225), but the secretory functions of MSCs might still be adequate. Interestingly, the death or trapping of therapeutic MSCs might still be advantageous as dead and apoptotic MSCs have been shown to have a potential immunomodulatory function (202). These apoptotic MSCs could induce IDO production by phagocytes helping immunosuppression (201), but it would be interesting to investigate further how apoptotic MSCs could control immune responses and how to exploit these cells for optimum therapies.

Overall, the legitimacy of MSC therapies for RA and SLE is currently increasing with various options for using different MSC types and administration approaches. However, future studies should focus on understanding why intrinsic/*in vivo* MSCs fail to function properly in autoimmune joint diseases, and how MSCs are being affected by chronic inflammatory milieu (226). These investigations would potentially help to introduce new therapeutic targets helping to correct the autoimmune reactions. MSC-based therapy in autoimmune joint disease could be further validated by having phase 3 clinical trials with large patient cohorts. More clinical studies are needed to directly compare the source and type of MSCs. While donor age appeared not affecting MSC functions (205), other factors such as the donor's disease, MSC culture conditions and mode of application should be also investigated for effect on autoimmune arthritis therapy.

## Author Contributions

JJE-J wrote the main parts of the first draft of the manuscript, prepared the figure, and updated all subsequent manuscript versions. YE-S helped with draft preparation and the tables. DM contributed to draft preparation and feedback on parts related to clinical aspects. EJ contributed into the draft preparation, provided the feedback on all sections, and wrote some parts related to exosome therapies. All authors contributed toward the design of the review, planning the sections, reviewed and made changes on the final draft, and agreed about the content.

## Conflict of Interest

The authors declare that the research was conducted in the absence of any commercial or financial relationships that could be construed as a potential conflict of interest.
